# Supplementary figures and images for: Emergence of microbial diversity due to cross-feeding interactions in a spatial model of gut microbial metabolism
Source: BMC Syst Biol. 2017 May 16;11:56. doi: 10.1186/s12918-017-0430-4 (PMC5434578; doi:10.1186/s12918-017-0430-4)

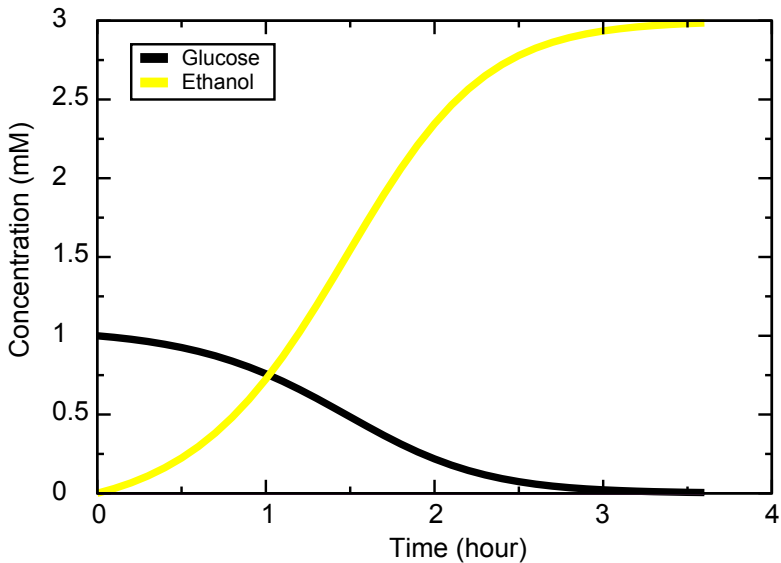

Supplement: Supplementary file 1 — Figure S1. Simulation of the non-spatial, extended L. plantarum model using standard flux-balance analysis (FBA). Metabolite dynamics over time. The simulation is initialized with a pulse of glucose. Note that with standard FBA all 1000 cells behave identically, because the crowding coefficients are not used. (PDF 93 kb) [file 12918_2017_430_MOESM1_ESM.pdf]

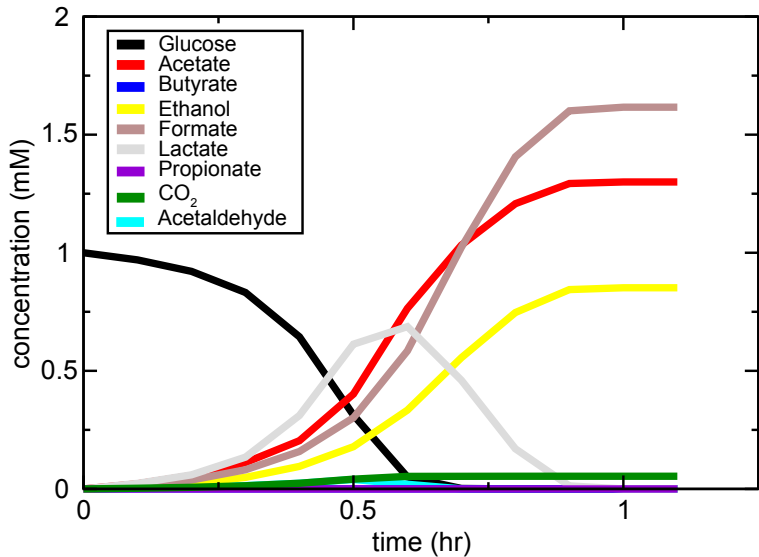

Supplement: Supplementary file 2 — Figure S2. Simulation of the non-spatial, standard L. plantarum model using flux-balance analysis with molecular crowding (FBAwMC). Metabolite dynamics over time. The simulation is initialized with a pulse of glucose. (PDF 105 kb) [file 12918_2017_430_MOESM2_ESM.pdf]

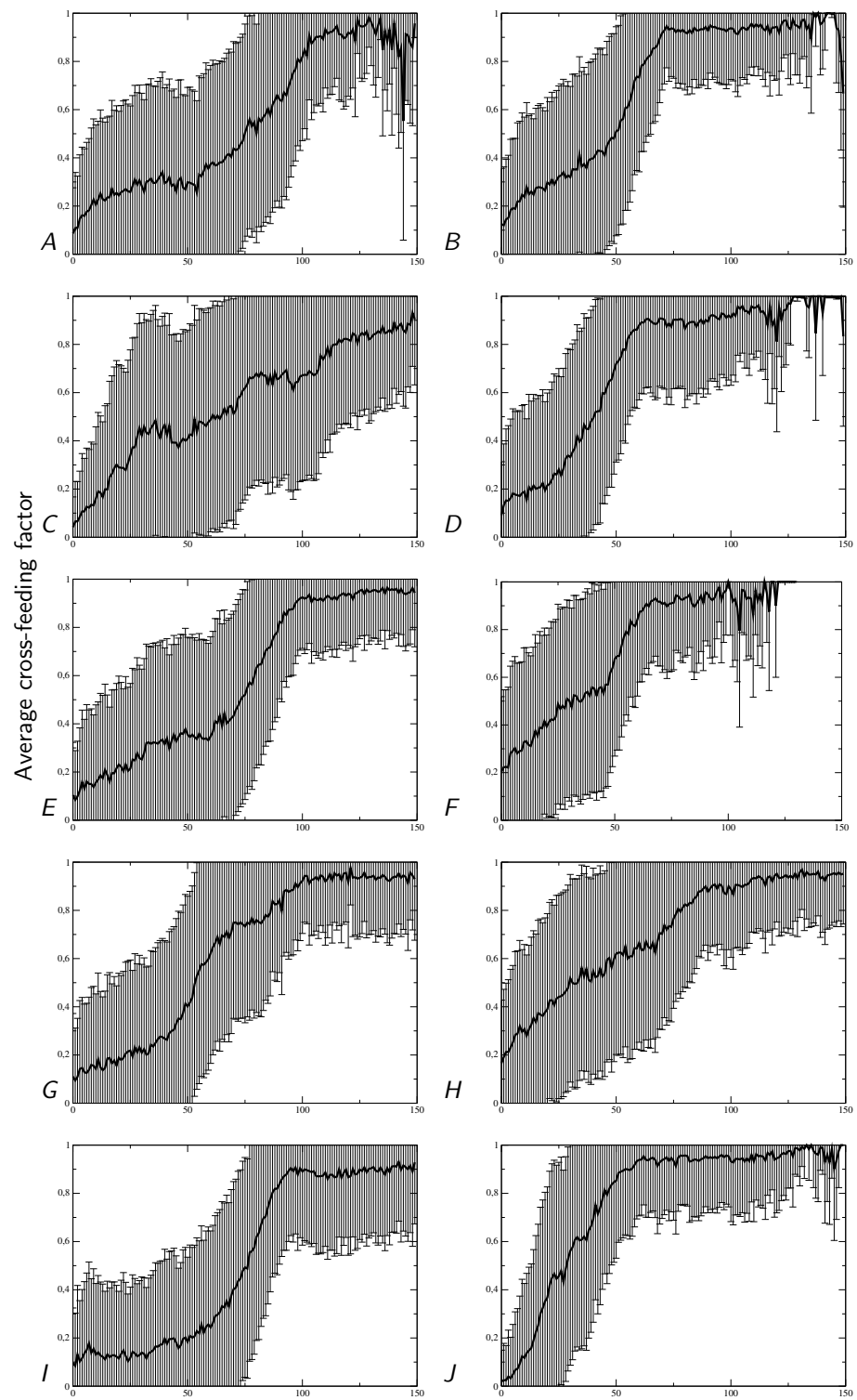

Proximal/distal position in the colon (pixel)

Supplement: Supplementary file 3 — Figure S3. Population average and standard deviation of the cross-feeding factor C i as a function of the position in the colon for all n=10 runs. The averages and standard deviation are over the vertical dimension and are calculated over the final part of the simulation, from 3500 h until 4000 h. (PDF 259 kb) [file 12918_2017_430_MOESM3_ESM.pdf]

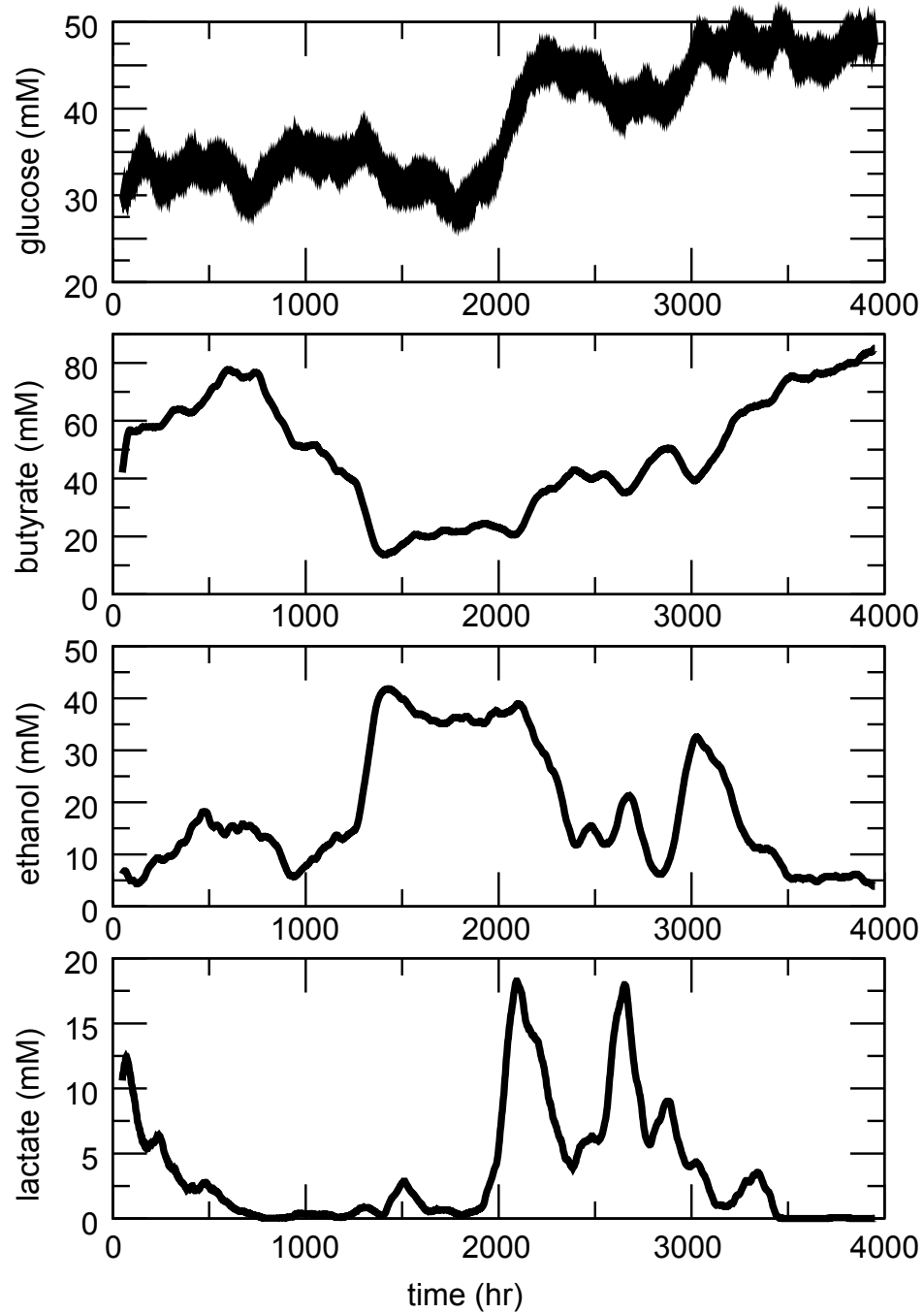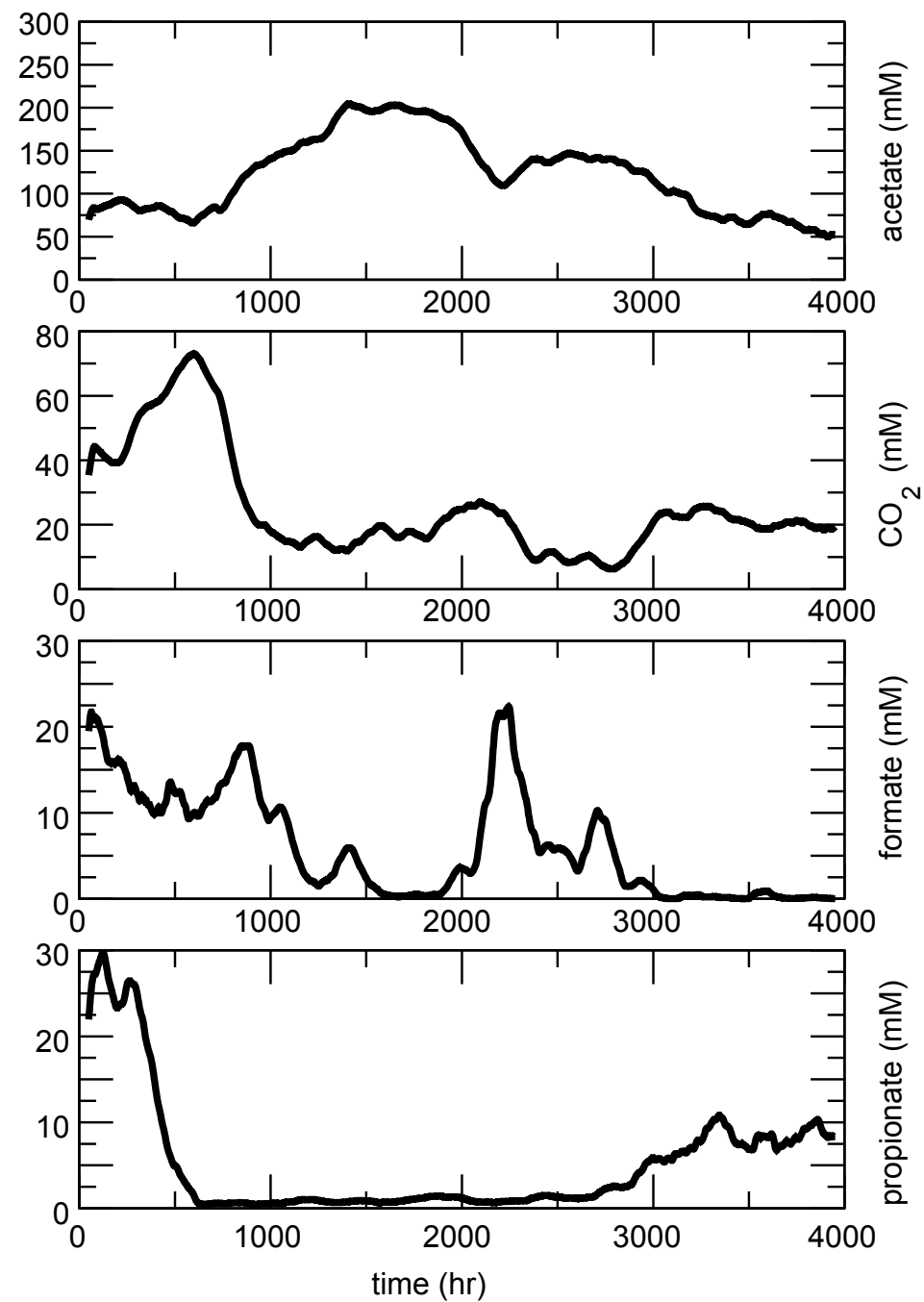

Supplement: Supplementary file 4 — Figure S4. Population averages of the metabolite concentrations over evolutionary time of the simulation in Fig. 5. (PDF 422 kb) [file 12918_2017_430_MOESM4_ESM.pdf]

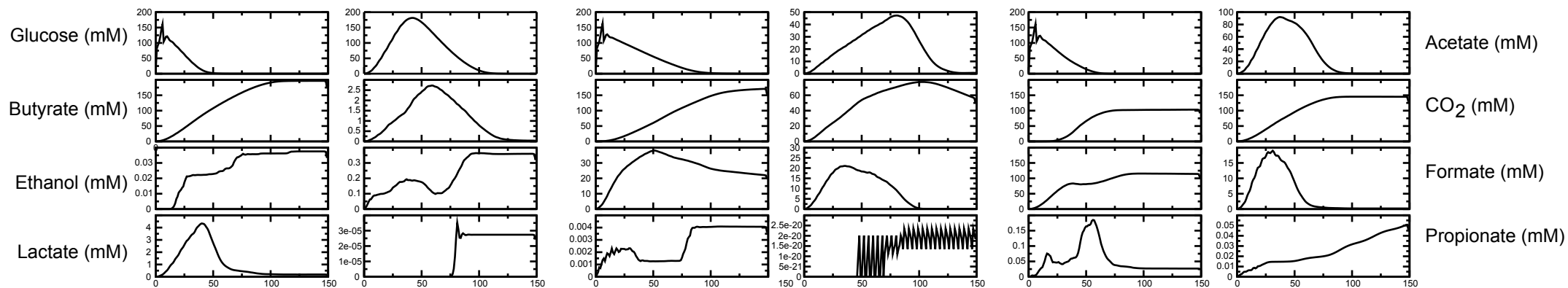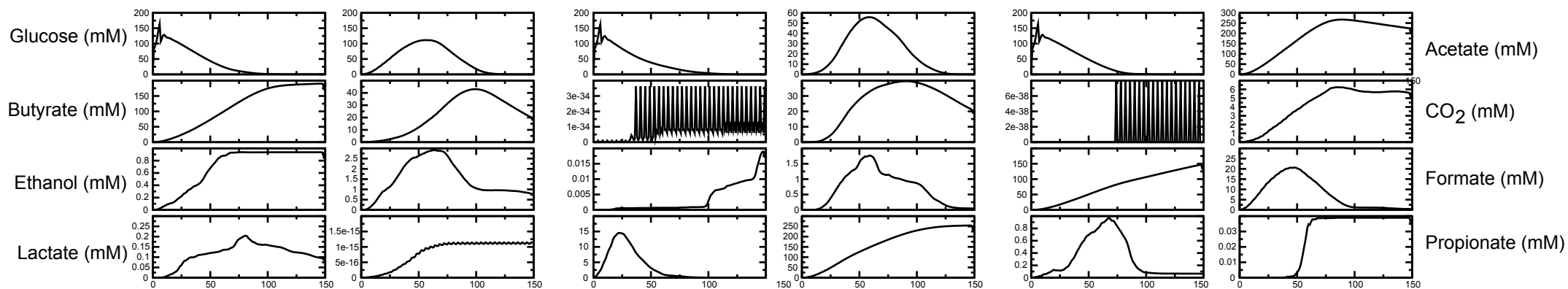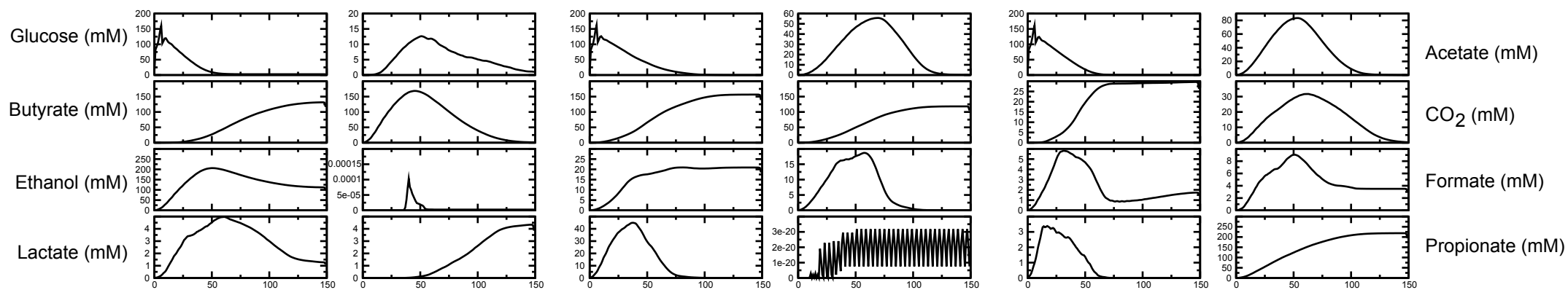

Position in colon (pixels)

Position in colon (pixels)

Position in colon (pixels)

Supplement: Supplementary file 5 — Figure S5. Average metabolite concentraties along the tube for all n=10 simulations. The averages are taken over the second half of the simulations, from 2000 h to 4000 h. (PDF 462 kb) [file 12918_2017_430_MOESM5_ESM.pdf]
